# Climate change and the aquatic continuum: A cyanobacterial comeback story

**DOI:** 10.1111/1758-2229.13122

**Published:** 2022-09-12

**Authors:** Brittany N. Zepernick, Steven W. Wilhelm, George S. Bullerjahn, Hans W. Paerl

**Affiliations:** ^1^ Department of Microbiology The University of Tennessee Knoxville Knoxville Tennessee USA; ^2^ NIEHS/NSF Great Lakes Center for Fresh Waters and Human Health Bowling Green State University Bowling Green Ohio USA; ^3^ Institute of Marine Sciences University of North Carolina at Chapel Hill Morehead City North Carolina USA

## Abstract

Billions of years ago, the Earth's waters were dominated by cyanobacteria. These microbes amassed to such formidable numbers, they ushered in a new era—starting with the Great Oxidation Event—fuelled by oxygenic photosynthesis. Throughout the following eon, cyanobacteria ceded portions of their global aerobic power to new photoautotrophs with the rise of eukaryotes (i.e. algae and higher plants), which co‐existed with cyanobacteria in aquatic ecosystems. Yet while cyanobacteria's ecological success story is one of the most notorious within our planet's biogeochemical history, scientists to this day still seek to unlock the secrets of their triumph. Now, the Anthropocene has ushered in a new era fuelled by excessive nutrient inputs and greenhouse gas emissions, which are again reshaping the Earth's biomes. In response, we are experiencing an increase in global cyanobacterial bloom distribution, duration, and frequency, leading to unbalanced, and in many instances degraded, ecosystems. A critical component of the cyanobacterial resurgence is the freshwater‐marine continuum: which serves to transport blooms, and the toxins they produce, on the premise that “*water flows downhill*”. Here, we identify drivers contributing to the cyanobacterial comeback and discuss future implications in the context of environmental and human health along the aquatic continuum. This Minireview addresses the overlooked problem of the freshwater to marine continuum and the effects of nutrients and toxic cyanobacterial blooms moving along these waters. Marine and freshwater research have historically been conducted in isolation and independently of one another. Yet, this approach fails to account for the interchangeable transit of nutrients and biology through and between these freshwater and marine systems, a phenomenon that is becoming a major problem around the globe. This Minireview highlights what we know and the challenges that lie ahead.

## INTRODUCTION

**FIGURE 1:**
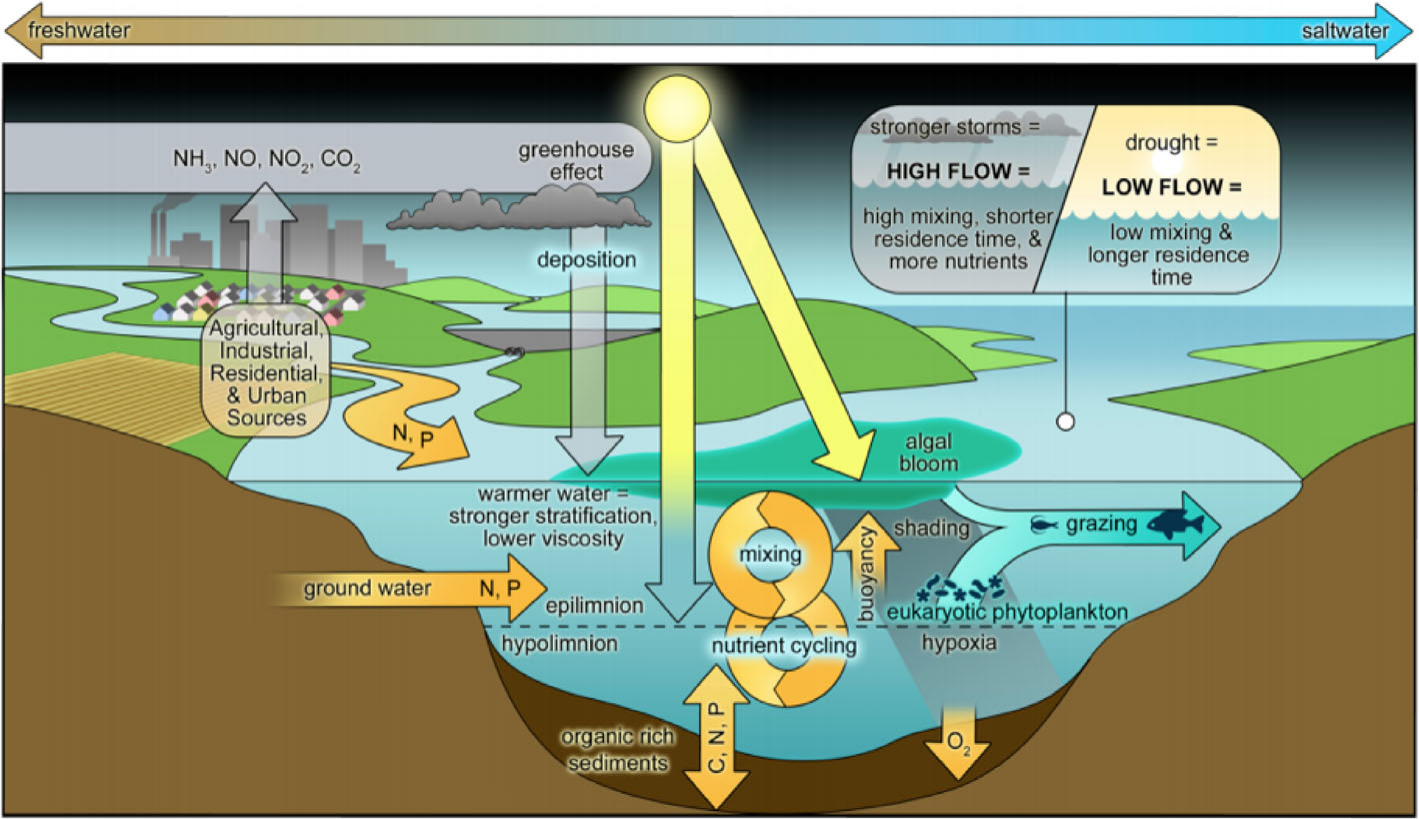
Diagram showing the interactive environmental controls on CyanoHABs along the freshwater‐marine continuum. Included are external (watershed and airshed) nutrient (nitrogen and phosphorus) inputs and internal nutrient cycling, hydrologic (freshwater discharge and its linkage to residence time) and physical (water column vertical mixing and irradiance) controls as well as linkages to oxygen cycling and food web interactions. *Source*: Figure adapted from Paerl, H.W. Toxins 10(2), 76 (2018). doi:10.3390/toxins10020076

The connections between freshwater sources, the receiving waters of rivers, estuaries, and the coastal ocean are rooted in a simple principle: *water flows downhill*. We are entering a juncture of the Anthropocene and global climatic change, with the combined effect being unprecedented pressures on ecosystems and human health (Masson‐Delmotte et al., 2018). Excessive nutrient inputs are accelerating eutrophication, with negative implications for water quality and its safe use along the freshwater‐marine continuum (Backer et al., 2010; Boesch et al., 2001; Bukaveckas et al., 2018; Schindler & Vallentyne, 2008; Wurtsbaugh et al., 2019) (Figure 1). Moreover, climatic changes across regional to global scales are exacerbating these pressures (Glibert, 2020; Havens & Paerl, 2015; Huisman et al., 2018; Moss et al., 2011). In particular, global warming has promoted harmful algal bloom taxa, especially toxic cyanobacteria (cyanoHABs), which prefer warmer temperatures (Huisman et al., 2018; Paerl & Huisman, 2008; Wells et al., 2015, 2020) and thrive under increasingly extreme oscillations in the wet/dry cycle (Gobler, 2020; Havens et al., 2016; Paerl et al., 2016, 2018). The purpose of this review is to describe the physiological characteristics of cyanobacteria, which make them ideally suited to persist and expand in an anthropogenically and climatically altered world. The irony that the very first oxygenic phototrophs on Earth (Schopf, 2000) have now successfully reclaimed their dominance has not escaped our attention.

### Ecophysiology and cyanobacterial adaptation to climate change across the continuum

For decades, there has been a concerted research effort to elucidate the factors responsible for cyanobacterial success (Hyenstrand et al., 1998; Paerl & Barnard, 2020; Stanier, 1977; Wilhelm et al., 2020). Studies have cited higher growth rates across environmental variables; including light intensity, temperature, residence time and water column stratification, as major drivers of cyanobacterial proliferation (Carey et al., 2012; Mur et al., 1999; Paerl & Huisman, 2008; Zahra et al., 2020). In addition, CO_2_ concentrating mechanisms (CCMs) (Burnap et al., 2015; Price et al., 2008; Sandrini et al., 2016), gas vesicle‐mediated buoyancy regulation (Brookes et al., 2000; Ganf & Oliver, 1982; Lürling et al., 2013) and nitrogen (N_2_) fixation (Zehr & Paerl, 2008) have been identified as contributors to their historical distribution and dominance. Yet, while many of these competitive attributes have been well‐studied within past and present contexts (Huisman et al., 2018; Steffen, Belisle, et al., 2014; Whitton & Potts, 2012), it remains to be determined how these qualities will serve as a benefit (or detriment) to cyanobacteria in the face of climate change along the freshwater‐marine continuum. In addition, the potential synergistic effects of these variables and their potential to promote cyanoHABs have yet to be fully ascertained. While these underlying mechanisms are the subject of current focus, the research and management communities generally agree that climatic changes (i.e. warming, more extreme wet/dry cycles, etc.) are leading to cyanobacterial proliferation. CyanoHABs are predicted to increase in distribution, duration and frequency across both the continuum (Paerl et al., 2018; Paul, 2008) and the globe (Harke et al., 2016; Huisman et al., 2018). In summary, we are observing a rebirth of conditions deemed favourable for cyanobacterial ecological success and dominance—conditions brought about by human activities.

Climate change is bringing about warmer temperatures, leading to increased thermal stratification of water columns (Hallegraeff, 2010; Kraemer et al., 2015). Prior studies indicate that when combined, higher temperatures and stratification synergistically favour cyanobacterial dominance (Joehnk et al., 2008; Paerl & Huisman, 2008; Wagner & Adrian, 2009). Further, as temperatures rise, the phytoplankton groups exhibiting the highest growth rates and abundances are transitioning from diatoms to cyanobacteria across aquatic ecosystems (Canale & Vogel, 1974; Ke et al., 2008). For example, the cyanoHAB genus *Microcystis* spp. has optimal growth temperatures ranging from 27.5 (You et al., 2018) to 32°C (Van Der Westhuizen & Eloff, 1985), with additional cyanoHAB taxa exhibiting even higher optima (Huisman et al., 2018). Further, cyanoHAB taxa such as *Microcystis* spp, *Dolichospermum* spp., and *Nodularia spumigena* have been found to reach peak annual abundances during maximum summer temperatures across various temperate lakes (Bertos‐Fortis et al., 2016; Davis et al., 2009; Wagner & Adrian, 2009). In addition to climate change‐induced expansion of their ecological niche, cyanoHABs are adept at reshaping their immediate environment themselves (Paerl & Millie, 1996; Paerl & Pinckney, 1996), ensuring their survival at the detriment of other phytoplankton taxa. For example, it has been proposed that cyanobacterial blooms may locally increase water column temperatures via intense light absorption and heat entrapment (Ma et al., 2016; Paerl & Huisman, 2008). This positive feedback loop serves to benefit cyanoHABs in freshwater systems such as the Baltic Sea (Kahru et al., 1993), and Lake Ijsselmeer, Netherlands (Ibelings et al., 2003). Indeed, the affinity of cyanoHABs to proliferate in warm stratified waters, coupled with the ability to optimize water column temperature themselves, serves to enhance their ecological success in their expansion along the continuum.

The capacity of cyanobacteria to alter the local environment (Paerl, 1996) is further exemplified in the emerging field of lake basification. In contrast to ‘the other CO_2_ problem’ known as ocean acidification (Doney et al., 2009), lake basification occurs when dense algal blooms deplete CO_2_ in the water column during periods of vigorous photosynthesis, driving up the pH (Ji et al., 2020; Sandrini et al., 2016; Verspagen et al., 2014). The water column pH remained at a daily average of ~9.2 for a month during a 2015 *Microcystis* spp. bloom in Lake Erie, United States/Canada (Zepernick et al., 2021), with similar phenomena observed in Lake Taihu, China (Van Dam et al., 2018) and Kennemermeer, the Netherlands (Sandrini et al., 2016). While the aforementioned freshwater systems observe seasonal cyanoHAB‐induced basification events, other systems exhibit year‐round occurrences. For example, Lake Santa Olalla, Spain exhibited a dramatic mean pH of 9.5 throughout a 2 year period, with the high pH levels attributed to cyanobacterial dominance (Lopez‐Archilla et al., 2004). These systems offer researchers a glimpse into the cyano‐dominated future by exemplifying conditions (such as year‐round basification) that may coincide with extended cyanoHAB events. To profit from these water column perturbations, cyanobacteria deploy an adaptive response to these high pH (and CO_2_ limited) events by altering their buoyancy and forming surface blooms where CO_2_ can be directly intercepted from the atmosphere (Cui et al., 2016; Hunter et al., 2008; Paerl & Ustach, 1982). Cyanobacteria also have excellent CCMs under elevated pH conditions (Burnap et al., 2015; Coleman, 1991; Mangan & Brenner, 2014), affording them continued access to CO_2_ during active blooms using carbonic anhydrases and bicarbonate transporters (Kaplan et al., 1998; Kupriyanova & Pronina, 2011). While cyanoHABs have been studied in the context of elevated pH, there has been a growing emphasis regarding how these high pH, carbon‐limited conditions affect phytoplankton beyond cyanobacteria (Turner et al., 2021; Wilhelm et al., 2020; Zepernick et al., 2021). Alkaline pH levels benefit *Microcystis* spp. and establish a positive feedback loop for bloom maintenance (Krausfeldt et al., 2019; Tang et al., 2018); yet, these same conditions constrain this cyanobacterium's competitors (i.e. freshwater diatoms) who exhibit decreased Si deposition and growth (Zepernick et al., 2021). In summary, climate change is expanding the cyanobacterial ecological niche, and these organisms themselves alter the water column to their benefit; both of which serve to facilitate their ecological success across the continuum and the globe.

Yet, when the environmental conditions prove sub‐optimal, cyanobacteria have evolved mechanisms to evade potential stressors. For example, cyanobacteria are exceptionally good at scavenging, assimilating and storing nitrogen (N) and phosphorus (P) compounds (Blomqvist et al., 1994; Moisander et al., 2008; Paerl, 2014). These attributes provide a competitive advantage in planktonic and benthic communities. With regard to N, Hyenstrand et al. (1998) and Newell et al. (2019) have shown bloom‐forming taxa to have superior combined N uptake mechanisms compared to eukaryotic phototrophs, with a strong preference for reduced forms of N (i.e. NH_4_, urea). Such scavenging capabilities can come into play when bloom‐induced pH rises above the pKa of NH_4_^+^/NH_3_, resulting in losses of NH_3_ to the atmosphere. During summer bloom periods when inorganic forms of N may be drawn down to low levels, cyanoHABs are also able to take advantage of water column and sediment regenerated N (Hampel et al., 2019). Indeed, many cyanobacteria can rapidly vertically migrate throughout the water column to access nutrient‐rich anoxic waters where reduced N regeneration products are plentiful. Also, cyanobacteria store cellular N in N‐rich phycobilins (phycocyanin, phycoerythrin) and cyanophycin, ensuring continuous cellular supplies (Grossman et al., 1993; Mackerras et al., 1990). Numerous cyanoHAB genera (i.e. *Aphanizomenon, Dolichospermum*, *Cylindrospermopsis*, *Nodularia*) are capable of fixing atmospheric N_2_ to NH_3_, further ensuring access to biologically available N when combined N sources are depleted. With regard to P, cyanoHABs similarly possess highly efficient uptake mechanisms, and when combined with the ability to store cellular P as polyphosphates, this ensures P availability when ambient supplies are low (Healy, 1982; Paerl, 2014). Overall, cyanoHABs possess dynamic, high‐affinity nutrient uptake mechanisms and formidable storage strategies, which facilitate their survival as they navigate the continuum.

Finally, when discussing the ecophysiology of cyanoHABs and bloom biomass, one must not forget the attending microbial community, or ‘phycosphere’, which plays a role in promoting bloom establishment, persistence and decline (Pound et al., 2021). Indeed, recent research has indicated certain heterotrophic constituents of the microbial community, such as α‐proteobacteria *Phenylobacterium*, have a potential role in facilitating the dominance of toxic *Microcystis* species in Lake Taihu, China and late‐stage bloom maintenance (Huertas Romera & Mallén Ponce, 2021; Zuo et al., 2021). One particular emerging concern is evidence that blooms may provide a suitable environment for multidrug‐resistant pathogens and the exchange of antibiotic resistance genes (Wang et al., 2020; Zhang et al., 2020). Consequently, the ecology of bloom‐forming cyanobacteria can pose threats across the continuum, which extend beyond hypoxia, basification events or the production of toxic metabolites.

### Toxicity: The harm that earns the sobriquet ‘cyanoHABs’

An algal bloom is not a monoculture, and several cyanobacterial genotypes of related taxa often coexist within a bloom (Reynolds, 2006). Many cyanobacteria produce secondary metabolites that can be toxic to animals, including humans (Paerl & Otten, 2013), and shift in toxin production and cyanobacterial taxa can occur rapidly during the course of a bloom season (Bukaveckas et al., 2018; Steffen, Zhu, et al., 2014). Currently, it is very difficult to predict the degree to which a bloom may change its composition and toxicity over time. However, there are several factors to consider regarding cyanotoxin production. Increased N availability has been shown to yield increased production of N‐rich cyanotoxins such as microcystins (Davis et al., 2015), with N speciation (NO_3_^−^, NH_4_^+^ and urea) influencing microcystin congener composition in some studies (Krausfeldt et al., 2019, 2020; Monchamp et al., 2014; Puddick et al., 2016). Lower temperatures also favour increased cellular microcystin quota, potentially yielding more toxic blooms during cooler periods (Martin et al., 2020; Peng et al., 2018). This latter point is important considering a changing climate also means more episodic storms and floods, which not only introduce growth‐favouring nutrients but also drop temperatures leading to short‐term spikes in toxin production.

Since cyanotoxins are intracellular metabolites, the risk to human exposure in drinking water can often be mitigated by the removal of bloom biomass via filtration and flocculation. However, lysis of cyanobacteria will release toxins into the dissolved phase, resulting in cyanotoxin contamination requiring aggressive and more costly water treatment protocols. Factors contributing to dissolved toxin release include salinity, which will increase along the freshwater‐estuarine continuum (Tonk et al., 2007) and cyanophage activity (McKindles et al., 2020; Steffen et al., 2017). Given that salinity has also been shown to influence a shift from phage lysogeny to a lytic state in environmental *Microcystis* spp. populations (Stough et al., 2017), salinity, nutrient and temperature gradients along the continuum provide multiple mechanisms to increase human exposure to cyanotoxins. In summary, climate change serves to exacerbate cyanoHAB toxin distribution, transport, and exposure across the continuum.

### Future mitigation of cyanobacterial HABs in a changing climate

Unfortunately, we cannot easily mitigate climatic changes taking place, although significantly reducing greenhouse gas emissions should remain a high priority for the long‐term protection of the Earth's resources (Masson‐Delmotte et al., 2018). While emerging research indicates a potential for future biotically based mitigation strategies (Huertas Romera & Mallén Ponce, 2021; Pal et al., 2020; Zuo et al., 2021), the development and implementation of such mitigation methods remains to be fully ascertained to date. Thus, the primary strategy applicable to controlling cyanoHABs in aquatic ecosystems is the immediate and aggressive reduction of nutrient inputs (Boesch et al., 2001; Conley et al., 2009; Paerl et al., 2020; Paerl & Barnard, 2020). For over a century, we have been aware of the benefits associated with nutrient loading reductions. Implementation of this strategy has been directly linked to water quality improvements in small aquatic systems (e.g. Lake Washington, USA and The Canadian Experimental Lakes, Canada) (Schindler & Vallentyne, 2008) and larger freshwater and brackish systems (e.g. Baltic Sea, Dutch Estuaries, Thames River, UK, Chesapeake Bay, Tampa Bay, USA) (Paerl et al., 2018; Paerl et al., 2020). Early efforts at tackling eutrophication in freshwater systems mainly focused on reducing P. This was largely due to observational and experimental work at that time, which pointed to P availability as controlling primary productivity and algal bloom formation (Schindler & Vallentyne, 2008), while it was shown that N availability controlled marine primary productivity (Nixon, 1995; Ryther & Dunstan, 1971).

Much has changed since the days of clear‐cut P or N limitation of eutrophication and HABs in both fresh‐ and salt‐water systems. More than a century of excessive anthropogenic P loading has led to a buildup or ‘legacy’ of P in the sediments and water columns of aquatic systems (Sharpley et al., 2013; Shatwell & Köhler, 2019). Phosphorus is not easy to remove or flush out of a water body because it is particle reactive, allowing it to be retained and internally cycled. In contrast, N has natural escape mechanisms, including volatilization of NH_3_ at high pH and denitrification to N_2_O and N_2_ (Salk et al., 2018), enabling losses of these N‐gases to the atmosphere.

This legacy P has shifted eutrophication nutrient controls from exclusive P to N&P co‐limitation and even temporal N limitation in many lakes, reservoirs, and rivers (Elser et al., 2007; Lewis et al., 2011; Paerl et al., 2016). Conversely, prolific use of chemical fertilizers and increased wastewater discharge has led to profound N enrichment, the impact of which has been to shift downstream riverine, estuarine and coastal water more towards N and P co‐limitation or even P limitation (Deng et al., 2021; Paerl et al., 2016; Sylvan et al., 2006). Nutrient reductions aimed at limiting cyanoHABs must take into consideration all these factors. These reductions can be applied on their own or in concert with other manipulative mitigation steps to reverse eutrophication, including dredging nutrient‐rich sediments, capping them, altering hydrological regimes by dam removal, and increasing water flushing, as well as short‐term ‘fixes’ aiming at temporarily arresting HABs (algaecides, sonication), and improving water column and sediment environmental conditions through artificial mixing and oxygenation (Paerl & Barnard, 2020). To be successful, these engineering‐oriented manipulations must be accompanied by comprehensive nutrient management plans. Moreover, these fixes need to be well‐founded in the available research literature; too often such remediation plans have unintended negative consequences (Hellweger et al., 2022). Nevertheless, a cohesive consensus on nutrient reduction strategies and mitigation tactics will be critical in combatting cyanoHAB expansion along the continuum.

Humans have had a profound impact on nutrient loading and nutrient limitation along the freshwater‐marine continuum (Paerl et al., 2016, 2018; Wurtsbaugh et al., 2019). Nutrient enrichment upstream can alter downstream nutrient limitation and productivity dynamics all the way to the coastal ocean (Paerl, 2009; Wurtsbaugh et al., 2019), with significant impacts on phytoplankton community composition (Glibert & Burford, 2017; Paerl et al., 2018). On the river basin scale, nutrient management aimed at controlling eutrophication upstream can have ramifications for downstream water quality, utilization and sustainability (Paerl et al., 2018). For example, excessive N loading resulting from high spring runoff and flooding in the Mississippi Basin can alter nutrient dynamics from N to P limitation in the receiving waters of the northern Gulf of Mexico (Sylvan et al., 2006). Furthermore, the ‘freshening’ associated with extreme rainfall and flooding events brings with it high nutrient loads, which can alter coastal habitats for HABs. One example is the proliferation of toxic cyanobacterial blooms (such as *Dolichospermum*), which were formerly confined to upstream lakes, estuaries and coastal Gulf of Mexico waters (Bargu et al., 2019). These rainfall events can also lead to episodic temperature changes that stimulate cyanobacterial taxa such as *Planktothrix*, which thrives at a broader temperature range (Davis et al., 2015; Post et al., 1985). Often, nutrient management aimed at controlling eutrophication and HABs upstream can have ramifications for water quality, water use and the sustainability of resources in downstream ecosystems. This calls for ‘scaling up’ regarding linking nutrient dynamics, human and climatic perturbations and altered hydrologic conditions on the continuum scale.

### The expansion of cyanobacterial HABs across the aquatic continuum

**FIGURE 2:**
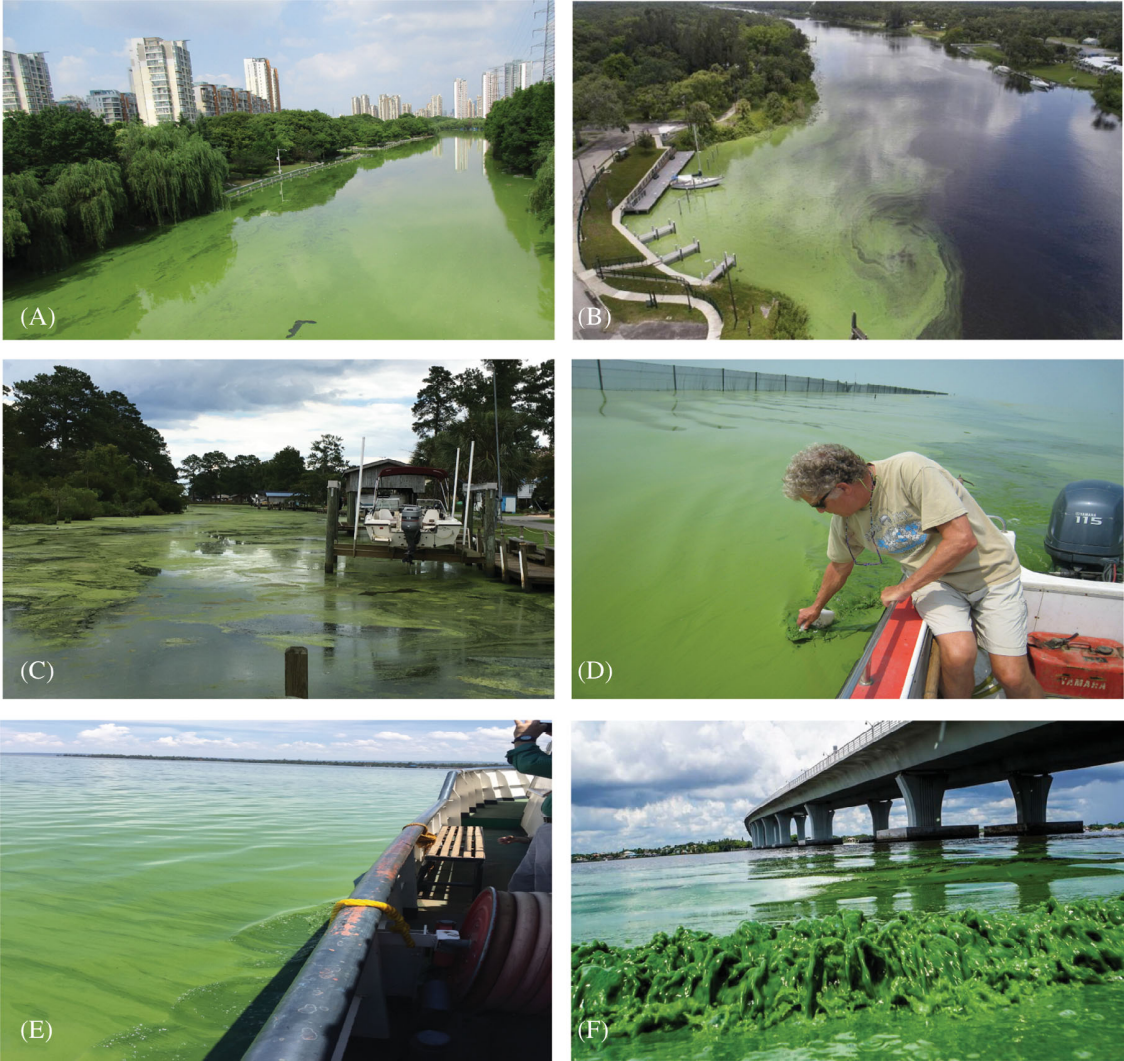
CyanoHABs observed along the freshwater‐marine continuum. (A) Liangxi River, a tributary of China's third largest freshwater lake, Taihu. *Source*: photo, Hans Paerl.(B) Caloosahatchee River draining Lake Okeechobee, Florida. *Source:* photo, Miami Herald.(C) Trent River, North Carolina, which discharges to the USA's second largest estuarine system, Albemarle‐Pamlico Sound. *Source*: photo, Hans Paerl. (D) Co‐author Hans Paerl, sampling CyanoHAB in Lake Taihu, China. *Source*: photo, Hai Xu and Hans Paerl. (E) Winam Gulf, Lake Victoria, Kenya. *Source*: photo, George Bullerjahn. (F) St. Lucie River entering the Jupiter inlet as a result of water released from Lake Okeechobee, Florida. *Source*: photo, Palm Beach Post/Associated Press

Freshwater HABs and their toxins are readily transported along the continuum into estuarine and coastal waters (Bukaveckas et al., 2018; Tatters et al., 2021) (Figure 2), where they can be freely incorporated by downstream shellfish species (Preece et al., 2015, 2017). Recent examples include (1) transport and proliferation of toxic cyanobacterial blooms (*Dolichospermum*) from upstream lakes, estuaries, and coastal N Gulf of Mexico waters; a situation that is aggravated by increased precipitation and floodwater discharges from the Mississippi watershed (Bargu et al., 2019). (2) Klamath Lake and the Klamath River, OR‐CA, where toxic cyanobacterial (*Microcystis*) blooms are transported to coastal Pacific Ocean waters (Genzoli & Kann, 2020). (3) The San Francisco Bay Delta, where cyanobacterial (*Microcystis*, *Dolichospermum*) blooms originating in upstream freshwater ‘tracts’ are transported into downstream saline San Francisco Bay (Ger et al., 2009; Lehman et al., 2010). (4) Cyanobacteria‐dominated Pinto Lake, which discharges toxic *Microcystis* into Monterey Bay, CA, where it is incorporated into the food chain via filter‐feeding clams that then adversely affect the health of local sea otter populations (Miller et al., 2010). Yet, perhaps most dramatic are dense toxic *Microcystis* blooms in the largest lake in the U.S. Southeast, Lake Okeechobee, FL that are transported via the Caloosahatchee river into estuarine waters on both the Atlantic (Indian River Lagoon) and Gulf of Mexico (Sanibel Estuary and Bay) coastlines (Metcalf et al., 2021; Rosen et al., 2018). This problem is exacerbated by recent upsurges in high rainfall tropical cyclones that have resulted in an overflow situation in Lake Okeechobee, causing the U.S. Army Corps of Engineers to release toxic blooms in both directions away from the lake. Additionally, there are a growing number of incipient, yet problematic releases of freshwater HABs into oligohaline estuaries. One example is the establishment and proliferation of cyanoHABs in brackish Albemarle Sound, NC a prime crab and shrimp fishing site and a major component of the second largest estuarine complex in the United States, the Albemarle‐Pamlico Sound, NC (Calandrino & Paerl, 2011). Lastly, climate change and lake warming have resulted in blooms forming where cyanoHABs were never previously thought to occur. Indeed, *Dolichospermum* sp. blooms now occur in cold (but warming), oligotrophic Lake Superior (Sterner et al., 2020). In summary, the cyanoHAB ‘colonization’ of these novel areas across the continuum is already becoming evident, with climate change serving to facilitate additional expansion.

## CONCLUDING REMARKS

To thwart the anthropogenically driven resurgence of the cyanobacteria, a broad understanding of cyanobacterial ecophysiology, environmental longevity, and continuum transference is paramount. Additionally, while nutrient reduction strategies have been regionally implemented with success, there is a need for a comprehensive mitigation strategy across the continuum, which includes solutions to both cyanoHAB transfer and the toxins which they produce. In making these considerations, we must remain cognizant of the fact that ‘*water runs downhill*’, and thus a solution to one region's problem can quickly become the problem of another's downstream. Nevertheless, what we do know is climate change serves to re‐establish cyanobacterial dominance, with recent evidence demonstrating their comeback is already well underway. The literature demonstrates successful mitigation protocols can be developed both regionally and globally, if supported by sound science. Yet, the question remains: are we prepared to handle the oncoming threat?

## AUTHOR CONTRIBUTIONS

All authors contributed to the drafting and final version of the manuscript.

## CONFLICT OF INTEREST

The authors declare that the research was conducted in the absence of any commercial or financial relationships that could be construed as a potential conflict of interest.

## Data Availability

Data are available upon request from the authors.
